# DNA barcoding reveals a new record of *Potamogeton distinctus* (Potamogetonaceae) and its natural hybrids, *Potamogeton distinctus* × *Potamogeton nodosus* and *Potamogeton distinctus* × *Potamogeton wrightii* (*Potamogeton ×malainoides*) from Myanmar

**DOI:** 10.3897/BDJ.2.e1073

**Published:** 2014-02-28

**Authors:** Yu Ito, Norio Tanaka, Rachun Pooma, Nobuyuki Tanaka

**Affiliations:** † University of Canterbury, Christchurch, New Zealand; ‡ Tsukuba Botanical Garden, National Museum of Nature and Science, Tsukuba, Japan; § Forest Herbarium, Bangkok, Thailand; | Kochi Prefectural Makino Botanical Garden, Kochi, Japan

**Keywords:** DNA barcoding, flora, Myanmar, new record, *
Potamogeton
*

## Abstract

Indo-China floristic region is among the 34 richest floristic regions of the world, and its plant diversity is still under investigation. Here we report a new record of an aquatic plant, *Potamogeton distinctus*, from Myanmar, a part of the region, that is detected by means of DNA barcoding method. The molecular method further identified the other specimens as hybrids of *Potamogeton*: one is Potamogeton ×malainoides (*Potamogeton distinctus* × *Potamogeton wrightii*), and the other is *Potamogeton distinctus* × *Potamogeton nodosus*. The first of these was thus far genetically confirmed in China, but the parental combination of the hybrid in Myanmar was reciprocal to those reported from China. The second hybrid was also recorded from China, but the maternal lineage was revealed for the first time, in this case it was *Potamogeton distinctus*. The present study showed that 1) nrITS is useful to distinguish closely related *Potamogeton* species as well as hybrids among them and 2) *atpB*-*rbcL* has higher utility than other frequently used plastid DNA markers. We thus propose nrITS and *atpB*-*rbcL* as DNA barcoding markers for future *Potamogeton* studies.

## Introduction

With many Southeast Asian countries included, Indo-China is among the 34 richest floristic regions of the world (Van Dijk et al. 2004), and its plant diversity is still under investigation. In the region, Myanmar is one of the countries where the floristic work has been insufficiently carried out, so many new species or noteworthy plant collections are still being reported from that country (Tanaka 2005). In order to explore further diversity of the flora, the present study targeted plant species that have not been recorded from Myanmar but are widely recognized in its neighbouring countries, such as southern part of China and Thailand. A member of an large aquatic genus, *Potamogeton* L., *Potamogeton distinctus* A. Benn. is one of these species.

*Potamogeton distinctus* is among the broad-leaved long-petioled *Potamogeton* species that is widely distributed in East Asia and Southeast Asia, including the southern part of China and Thailand (Wiegleb 1990). Both the only floristic checklist of Myanmar and the first aquatic plants checklist of Myanmar do not include this species but lists morphologically similar other broad-leaved long-petioled species, *Potamogeton nodosus* Poir. and *Potamogeton wrightii* Morong (Kress et al. 2003, Ito and Barfod 2014). Whereas the other reported Potamogeton species from Myanmar can be easily distinguished from *Potamogeton distinctus*, e.g., by the shape of submerged or floating leaves, the two broad-leaved long-petioled *Potamogeton* can only be recognized with floral morphology because the reliable diagnostic character of *Potamogeton distinctus* is the flower with two carpels, which is four-carpellate in the other species (Wiegleb 1990); this characteristic, of course, could not be applied to non-flowering specimens, which many of *Potamogeton* collections from Myanmar are. This indicates that *Potamogeton distinctus* might be misidentifed as one of the other broad-leaved long-petioled *Potamogeton* species and thus overlooked in the flora.

*Potamogeton* is known to have aneuploidy, polyploidy, and hybridization (Les 1983). The different cytotypes, i.e., aneuploids and polyploids, are phylogenetically well clustered (Kaplan et al. 2013); hence no inter-specific taxonomic confusions occur by aneuploidy and polyploidy. On the other hand, the known numerous inter-specific hybrids may cause a confusion, because the hybrids are in most cases difficult to recognize solely based on morphological investigation (Les et al. 2009). In Myanmar, although no natural *Potamogeton* hybrids have been reported, among the listed nine species and two synomyous ones by Kress et al. (2003) or six species by Ito and Barfod (2014) are *Potamogeton nodosus* and *Potamogeton wrightii*, both of which are known to hybridize with *Potamogeton distinctus* in China (Du et al. 2010). Hence, taxonomic confusion might have occurred in the inventory of *Potamogeton distinctus* in Myanmar with apparent hybridiｚations with the other broad-leaved long-petioled *Potamogeton* species.

In such cases, analysis of plant DNA sequence data can provide an effective method, that is known as DNA barcoding (e.g., Chase et al. 2005, Kress et al. 2005, CBOL Plant Working Group 2009). This method was initially launched to target diverse plant groups with universal DNA markers, e.g., flowering plants (*trnH*-*psbA* and the multi-copy internal transcribed spacer of nuclear ribosomal DNA (nrITS): Kress et al. 2005), vascular plants (*matK* + *rbcL*: Saarela et al. 2013), or land plants (*matK* + *rbcL*: CBOL Plant Working Group 2009). Recently the applications of DNA barcoding shifted to target narrow plant groups with respective unique DNA markers, e.g., *Compsoneura* of Myristicaceae (*matK* + *trnH*-*psbA*: Newmaster et al. 2008), Combretaceae (*trnH*-*psbA*: Gere et al. 2013), Hymenophyllaceae (*rbcL*, *trnSGG*, and *trnH*-*psbA*: Nitta 2008), mosses (*trnH*-*psbA* and *rps4*: Liu et al. 2001), *Phoenix* of Arecaceae (*psbZ*-*trnfM*: Ballardini et al. 2013), or *Viburnum* of Adoxaceae (*trnH*-*psbA* and nrITS: Clement and Donoghue 2012). Barcoding studies occasionally lead to discoveries of new records of plants species from surveyed regions (Liu et al. 2001, Nitta 2008). In *Potamogeton*, four candidate DNA markers were tested and of these nrITS was proposed as the most useful DNA barcoding marker (Du et al. 2011). The nuclear DNA marker would be applicable for any purposes because almost all *Potamogeton* species as well as hybrids were distinguishable with this marker (e.g., Du et al. 2010, Ito and Tanaka 2013, Kaplan and Fehrer 2011, Les et al. 2009). Meanwhile, in order to understand precisely the apparent hybridization events, plastid DNA (ptDNA) markers should be simultaneously applied, so that maternal phylogenetic information would be available (Kaplan and Fehrer 2006). The candidate markers included *atpB*-*rbcL* (Ito et al. 2007), *rpl20*-*rps12* (Kaplan and Fehrer 2011), and *trnT*-*trnL*, *trnL*, *trnL*-*trnF* (Ito and Tanaka 2013).

The present study aimed to assess the potential occurrence of *Potamogeton distinctus* and its inter-specific hybrids, if any are present, in Myanmar. To do so, we applied a taxon-specific DNA barcoding method. First, in order to evaluate the utility of selected DNA barcoding markers, we performed simultaneous molecular phylogenetic analyses based on a sample set of precisely identified broad-leaved long-petioled *Potamogeton* specimens, occasionally suplimented with some GenBank accessions. Then, using the DNA barcoding markers, we assigned broad-leaved long-petioled *Potamogeton* specimens from Myanmar, which could not be identified by morphology due to either the lack of diagnostic floral characters or intermediate vegetative morphology or both. The resulting molecular insights of broad-leaved long-petioled *Potamogeton* species in Myanmar will be used to document a new record of *Potamogeton* species for the flora of Myanmar, to discuss the origin and the evolution of hybrids of *Potamogeton* in Myanmar, and to propose DNA barcoding markers for future *Potamogeton* studies.

## Materials and methods

### Plant material

We carried out a field expedition to Myanmar in 2008 and collected four relevant specimens, i.e., broad-leaved long-petioled *Potamogeton* specimens, including three non-flowering and one flowering ones in Shan state (Table 1). None of the specimens could be morphologically identified as any of three broad-leaved long-petioled *Potamogeton* species potentially distributed in Myanmar (*Potamogeton distinctus*, *Potamogeton nodosus*, and *Potamogeton wrightii*) due to either the lack of diagnostic floral characters or intermediate vegetative morphology or both. The morphological characters of the unidentified specimens were summarized to facilitate comparison with the three *Potamogeton* species (Table 1).

To evaluate the utility of selected DNA barcoding markers through performing molecular phylogenetic analyses, comparative materials of *Potamogeton distinctus*, *Potamogeton nodosus*, and *Potamogeton wrightii* were collected in Japan, Mexico, and Thailand (Table 2). As we failed to collect hybrids of *Potamogeton distinctus*, the nrITS data sets of two *Potamogeton* hybrids were obtained from GenBank: Potamogeton ×malainoides Miki (*Potamogeton distinctus* × *Potamogeton wrightii*) and *Potamogeton distinctus* × *Potamogeton nodosus* (Du et al. 2010). Besides, two outgroup species were selected following Lindqvist et al. (2006) and included into the sample set; those were *Potamogeton lucens* L. and *Potamogeton perfoliatus* L. Note that four out of the six comparative materials were previously used for molecular phylogenetic analyses (Ito and Tanaka 2013).

The voucher specimens are retained in either of the following herbaria: BKF; MBK; RAF; TI; TNS. Those of Du et al. (2010) are kept in HIB. Sequences were deposited at the DNA Data Bank of Japan (DDBJ) and their accession numbers and voucher information are given in Table 2.

### DNA extraction, amplification and sequencing

For the newly obtained samples, total genomic DNA was extracted and sequencing of five plastid regions was performed using the procedure outlined by Ito et al. (2010). For the sequencing, previously used accessions were occasionally involved. We selected the following DNA regions that were used in previous molecular studies of *Potamogeton* as DNA barcoding markers: *atpB*-*rbcL* (Ito et al. 2007), *rpl20*-*rps12* (Kaplan and Fehrer 2011), *trnT*-*trnL*, *trnL*, *trnL*-*trnF* (Ito and Tanaka 2013, Zhang et al. 2008), and nrITS (e.g., Du et al. 2010, Ito and Tanaka 2013, Kaplan and Fehrer 2011, Les et al. 2009). The *atpB-rbcL* (seven samples), *rpl20*-*rps12* (nine), *trnT*-*trnL* (four), *trnL* (five), and *trnL*-*trnF* (five) regions of chloroplast DNA were amplified and directly sequenced using primers atpB-2F and rbcL-2R (Manen et al. 1994) for *atpB-rbcL* (779–787 bp), rpl-20 and 5'-rps-12 (Hamilton 1999) for *rpl20*-*rps12* (794 or 813 bp), and Po-trnT2F (Ito and Tanaka 2013) and “b” (Taberlet et al. 1991) for *trnT*-*trnL* (807–809 bp), “c” and “d” (Taberlet et al. 1991) for *trnL* intron (593 bp), and “e” and “f” (Taberlet et al. 1991) for *trnL-trnF* (403 bp). Note that *trnT*-*trnL* was missing from *Potamogeton* sp. (N. Tanaka & al. 080662).

Sequences of the nrITS were obtained using primers ITS-4 and ITS-5 (Baldwin 1992) under the same conditions used for the *phyB* amplification in Ito et al. (2010). The total length was 713 bp. On direct sequencing of ten samples, overlapping double peaks were found at the same sites for complementary strands in the electropherograms. These products were cloned using a TOPO TA Cloning kit for Sequencing (Invitrogen, Carlsbad, California, USA). At least 16 clones per sample were chosen and their sequences were determined using the same procedure as that used in the first PCR followed by direct sequencing. For the cloned sequences, nucleotides that were not detected by direct sequencing were regarded as PCR errors.

### Data analysis

Sequences of the *atpB*-*rbcL*, *rpl20*-*rps12*, *trnT-trnL*, *trnL*, *trnL-trnF*, and nrITS regions were manually aligned using the simple indel coding method of Simmons and Ochoterena (2000). Gaps associated with mononucleotide repeat units were removed from consideration in the phylogenetic analysis because of problems related to homology assessment (Kelchner 2000) and because technical artifacts might be responsible for the variation (Clarke 2001). One representative sequence was used for accessions having the identical combined sequence.

Phylogenetic analyses were independently performed for data sets of ptDNA (*atpB*-*rbcL*, *rpl20*-*rps12*, *trnT-trnL*, *trnL*, *trnL*-*trnF*) and nrITS, respectively. Phylogenetic inference was performed using maximum parsimony (MP) in PAUP* 4.0b10 (Swofford 2002) and Bayesian inference (BI; Yang and Rannala 1997) in MrBayes 3.1.2 (Ronquist and Huelsenbeck 2003) as described by Ito and Tanaka (2013); the only differences were the best-fit model for BI analysis on ptDNA (F81) and nrITS (HKY). The Bayesian Markov Chain Monte Carlo algorithm was run for 1 million generations for both ptDNA and nrITS data sets. Four incrementally heated chains were used that started from random trees and sampled one out of every 100 generations. The first 25% of the sampled generations (250,000 generations for each data set, respectively) were discarded as burn-in, and the remaining trees were used to calculate a 50% majority-rule consensus tree and to determine posterior probabilities for branches. The data matrices and the MP trees are available from the TreeBASE (S14928).

## Taxon treatments

### Potamogeton distinctus

A. Benn.

#### Materials

- **Type status:** Other material. **Occurrence:** recordedBy: Y. Ito; **Location:** country: Myanmar; stateProvince: Shan; verbatimLocality: Yae Aye Kan; Kalaw; verbatimLatitude: 20 35 41 N; verbatimLongitude: 96 31 46 E; **Event:** eventDate: 26 Nov 2008; **Record Level:** collectionID: N. Tanaka & al. 080061; institutionCode: MBK, RAF, TI
- **Type status:** Other material. **Occurrence:** recordedBy: Y. Ito; **Location:** country: Myanmar; stateProvince: Shan; verbatimLocality: Nyaun Shwe; Inlay Lake; verbatimLatitude: 20 32 02 N; verbatimLongitude: 96 53 53 E; **Event:** eventDate: 3 Dec 2008; **Record Level:** collectionID: N. Tanaka & al. 080657; institutionCode: MBK, RAF, TI

#### Distribution

?Bhutan, China (nationwide), Korea, Japan, Myanmar, Nepal, ?Philippines, Thailand, ?Vietnam (modified from Wiegleb 1990).

#### Taxon discussion

*Potamogeton distinctus* shows a wide range of phenotypic plasticity, especially in leaf morphology. It seems to be that the two-carpellate flower, the diagnostic character of the species, is essential for precise morphological identification in the field; identification with vegetative morphology alone is to be avoided (see Discussion).

## Analysis

### Molecular phylogenetic analyses based on ptDNA and nrITS

**Figure 1.**
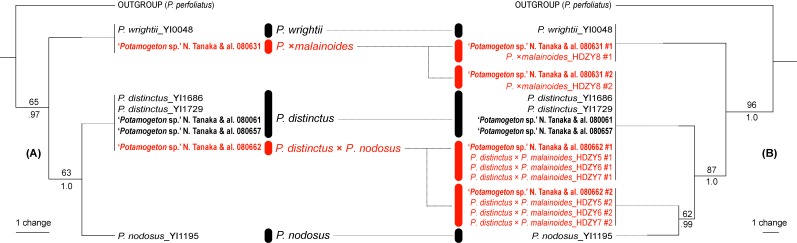
The most parsimonious trees of *Potamogeton* based on (A) the combined plastid DNA (*atpB*–*rbcL*, *rpl20*–*rps12*, *trnT–trnL*, *trnL*, *trnL*–*trnF*) sequences and (B) nrITS sequences. Each one of the two outgroups is trimmed to clarify ingroup phylogeny. ACCTRAN optimisation is used for branch length measures; terminals are aligned with dotted lines. Numbers above the branches indicate bootstrap support (BP) calculated in the maximum parsimony, and those below indicate Bayesian posterior probabilities (PP). Samples in regular and bold face indicate comparative ones and those from Myanmar, respectively. Some accessions in each tree represent multiple identical accessions. Note that some samples have heterogeneous nrITS copies; for these, the sequence pairs are named #1 and #2, respectively, and colored in red.

The length of the combined five ptDNA regions alignment containing ten accessions totaled 3456 bp, of which two characters were parsimony-informative. Based on this data set, one MP tree (tree length = 27 steps; consistency index = 1.0; retention index = 1.0) and a BI 50% consensus tree were obtained. These trees showed congruent phylogenetic relationships and thus only the MP tree is presented here (Fig. 1).

The length of nrITS alignment composed of 20 accessions totaled 645 bp, of which six characters were parsimony-informative. In the phylogenetic analysis of nrITS data set, one MP tree (tree length = 43 steps; consistency index = 1.0; retention index = 1.0) and a BI 50% consensus tree were obtained. These trees showed congruent phylogenetic relationships and thus only the MP tree is presented here (Fig. 1).

In both ptDNA and nrITS trees, the three morphologically closely related species were well differentiated from one another. With *Potamogeton lucens* and *Potamogeton perfoliatus* as outgroup, *Potamogeton wrightii* and the clade of *Potamogeton distinctus* and *Potamogeton nodosus* were clustered (63 MP bootstrap (BS), 1.0 BI posterior probability (PP) in ptDNA; 87 MP BS, 1.0 PP in nrITS). *Potamogeton nodosus* from Mexico and *Potamogeton nodosus*-related nrITS sequence of *Potamogeton distinctus* × *Potamogeton nodosus* HDZY5-7 showed variation, yet the two sequences were clustered each other (62 MP BS, 0.99 BI PP). GenBank accessions of Potamogeton ×malainoides and *Potamogeton distinctus* × *Potamogeton nodosus* (Du et al. 2010) have diverged heterogeneous nrITS sequences, and non-hybrid species have homogenous nrITS sequences (Fig. 1).

### DNA barcoding for broad-leaved long-petioled *Potamogeton* specimens from Myanmar

**Figure 2.**
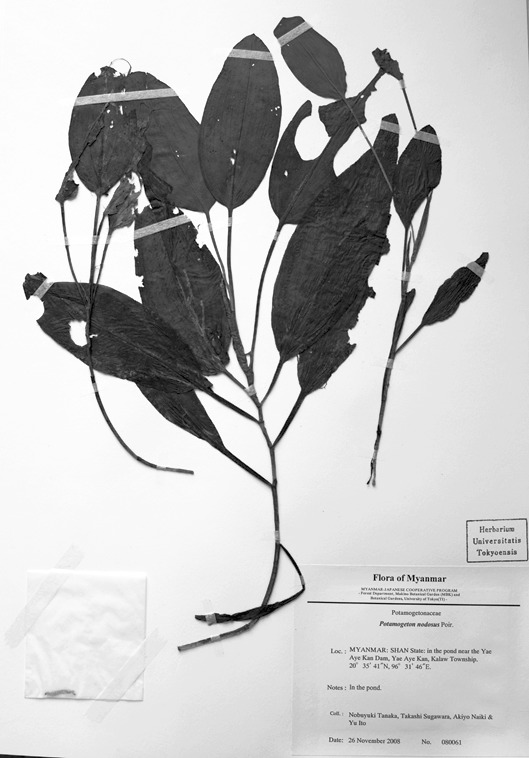
A voucher specimen of *Potamogeton distinctus* (N. Tanaka & al. 080061).

**Figure 3.**
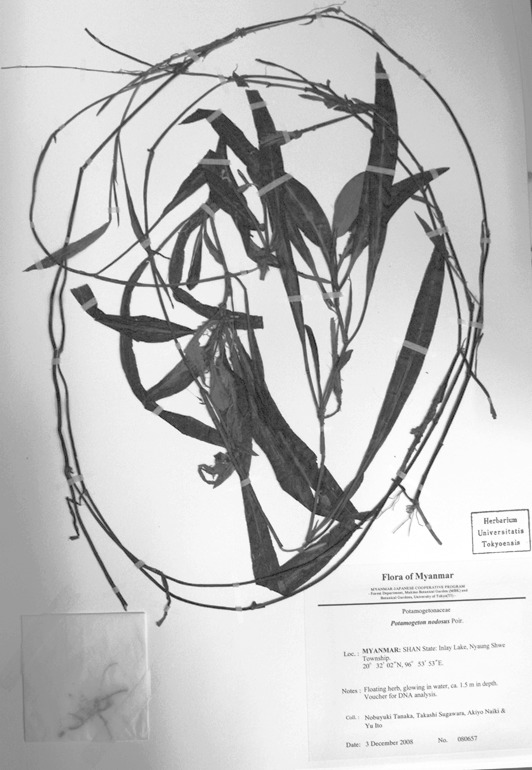
A voucher specimen of *Potamogeton distinctus* (N. Tanaka & al. 080657).

**Figure 4.**
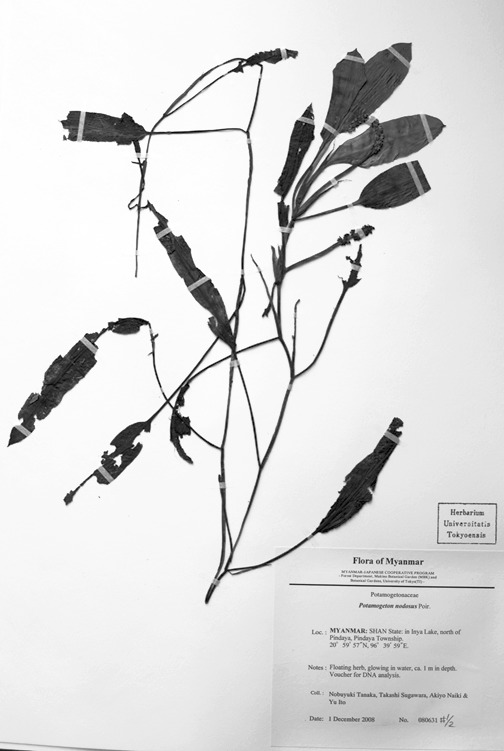
A voucher specimen of Potamogeton ×malainoides (N. Tanaka & al. 080631).

**Figure 5.**
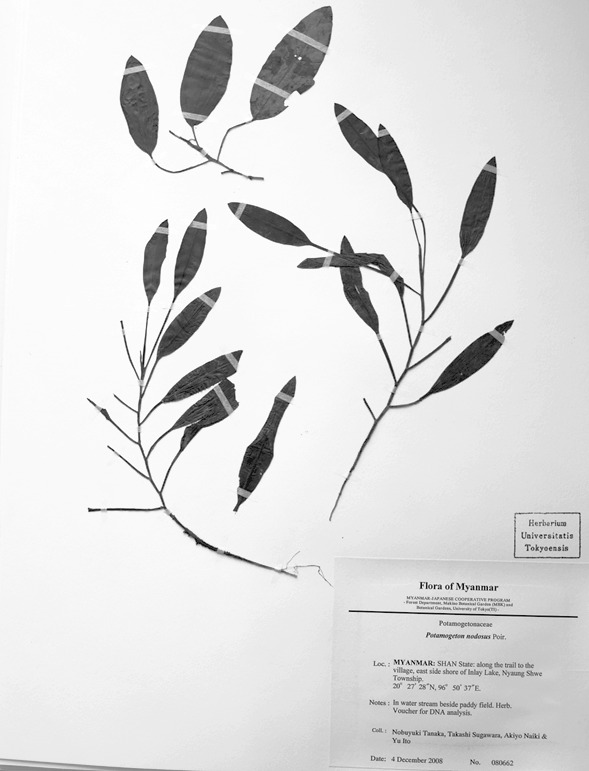
A voucher specimen of *Potamogeton distinctus* × *Potamogeton nodosus* (N. Tanaka & al. 080662).

Of the four broad-leaved long-petioled *Potamogeton* specimens from Myanmar, two were genetically identical to *Potamogeton distinctus* from Japan and Thailand (N. Tanaka & al. 080061, N. Tanaka & al. 080657; Figs 2, 3). Another specimen had *Potamogeton wrightii* haplotype and both of the heterogeneous nrITS sequences of Potamogeton ×malainoides (N. Tanaka & al. 080631; Fig. 4); the other of the remaining two exhibited *Potamogeton distinctus* haplotype and both of the heterogeneous nrITS sequences of *Potamogeton distinctus* × *Potamogeton nodosus* (N. Tanaka & al. 080662; Fig. 5).

### Utility of DNA barcoding markers for *Potamogeton* species

The combined five ptDNA regions were separately analyzed to facilitate the utility as individual DNA markers. The comparison included nrITS. Between the closely related species, *Potamogeton distinctus* and *Potamogeton nodosus*, where two nucleotide substitutions were observed in nrITS, *atpB*-*rbcL* exhibited one nucleotide substitution, while *trnT*-*trnL* showed a difference in mononucleotide repeat unit (Tables 3, 4). Among the three species, in which ten nucleotide substitutions were found in nrITS, *atpB*-*rbcL* included one length variation (indel) and two nucleotide substitutions; *trnT*-*trnL* region had two mononucleotide repeat units, in which repeat numbers are differed.

## Discussion

In order to assess the potential occurrence of *Potamogeton distinctus* and its hybrids, if any are present, in Myanmar, the present study applied a taxon-specific DNA barcoding method. The simultaneous molecular phylogenetic analyses successfully distinguished broad-leaved long-petioled *Potamogeton* species, *Potamogeton distinctus*, *Potamogeton nodosus*, and *Potamogeton wrightii*, as well as hybrids among them, Potamogeton ×malainoides (*Potamogeton distinctus* × *Potamogeton wrightii*) and *Potamogeton distinctus* × *Potamogeton nodosus* (Fig. 1). The obtained phylogeny is congruent with the nuclear 5S-NTS phylogeny of Lindqvist et al. (2006), the only molecular phylogeny that resolves the three *Potamogeton* species relationships. Below we will document a new record of *Potamogeton* species for the flora of Myanmar, discuss the origin and the evolution of hybrids of *Potamogeton* in Myanmar, and propose DNA barcoding markers for future *Potamogeton* studies.

### *Potamogeton nodosus*, a new record for the flora of Myanmar

Applying the comparative samples’ sequence data as DNA barcodes, the broad-leaved long-petioled *Potamogeton* specimens from Myanmar were genetically assigned. As a result, two out of four specimens were identified as *Potamogeton distinctus*, a widely distributed species in East Asia, Southeast Asia and the Pacific, including southern part of China and Thailand, but not in Myanmar (Wiegleb 1990). Here we document a new record for the flora of Myanmar.

### Hybridization among broad-leaved long-petioled *Potamogeton* species in Myanmar

The taxon-specific DNA barcoding also revealed two hybrids of *Potamogeton* in Myanmar, and among which was Potamogeton ×malainoides (*Potamogeton distinctus* × *Potamogeton wrightii*). This hybrid is known from China (Du et al. 2010), yet a difference is found between the Chinese and Myanmar cases in maternal lineage: Potamogeton ×malainoides from China has *Potamogeton distinctus* as a maternal parent (Du et al. 2010), but that from Myanmar has *Potamogeton wrightii* as a maternal parent. This kind of reciprocal hybridizations occasionally occur in *Potamogeton*, i.e., Potamogeton ×anguillanus, Potamogeton ×fluitans, Potamogeton ×inbaensis, Potamogeton ×lanceolatifolius, Potamogeton ×sudermanicus, and Potamogeton ×suecicus (reviewed in Ito and Tanaka 2013). In terms of morphology, Potamogeton ×malainoides in Myanmar showed both *Potamogeton distinctus* character, i.e., larger number of leaf veins, and that of *Potamogeton wrightii*, i.e., the acute to acuminate leaf tip (Table 1), and no major differences are found between the reciprocal hybrids (Du et al. 2010). In other cases of *Potamogeton* hybrids, reciprocal hybrids are partly distinguishable, e.g., reciprocal Potamogeton ×anguillanus shows no differences in morphology but exhibited differences in drought tolerance (Iida et al. 2007); Potamogeton ×inbaensis with different maternal lines is roughly distinguishable by leaf morphology (Amano et al. 2008).

The other hybrid of *Potamogeton* identified in Myanmar is *Potamogeton distinctus* × *Potamogeton nodosus*. This hybrid is also known from China, yet no maternal lineage was conclusively identified in the previous study (Du et al. 2010). The present study successfully identified *Potamogeton distinctus* as the maternal lineage of this hybrid for the first time. From the morphological point of view, it is difficult to evaluate the morphological intermediacy between the parental species as both species show large phenotypic plasticity in quantitative morphology, e.g., leaf petiole length.

### Utility of DNA barcoding markers for *Potamogeton* species

Du et al. (2011) reported that nrITS is the most useful marker for DNA barcoding of *Potamogeton*. The present study verified its utility by distinguishing three closely related species, *Potamogeton distinctus*, *Potamogeton nodosus*, and *Potamogeton wrightii*, as well as hybrids among them (Fig. 1, Table 3). Meanwhile, in order to understand hybridization events precisely, we simultaneously used plastid DNA markers, including those used in previous molecular studies, i.e., *atpB*-*rbcL* (Ito et al. 2007), *rpl20*-*rps12* (Kaplan and Fehrer 2011), and *trnT*-*trnL*, *trnL*, *trnL*-*trnF* (Ito and Tanaka 2013, Zhang et al. 2008). Given that *atpB*-*rbcL* showed higher utility than the others (Table 4), here we propose nrITS and *atpB*-*rbcL* as DNA barcoding markers for *Potamogeton* species. Note that *trnT-trnL* has similar resolution to distinguish closely related *Potamogeton* species, yet the differences are found only in mononucleotide repear units, which technical artifacts might be responsible for the variation (Clarke 2001).

The taxon-specific DNA barcoding method presented here will be applicable in elucidating further diversity of *Potamogeton* in other floras. With some modification on marker selection, this method will be also applicable for floras that focus on other taxa.

## Supplementary Material

XML Treatment for Potamogeton distinctus

## Figures and Tables

**Table 1.** Morphological comparison among the four *Potamogeton* specimens collected in Myanmar and three broad-leaved long-petioled *Potamogeton* species potentially distributed in Myanmar (a: Wiegleb 1990; b: Wiegleb and Kaplan 1998; c: Wiegleb 2002; d: Guo et al. 2010).

	taxon	*Potamogeton distinctus*	*Potamogeton nodosus*	*Potamogeton wrightii*	*Potamogeton* sp. (N. Tanaka & al. 080061)	*Potamogeton* sp. (N. Tanaka & al. 080657)	*Potamogeton* sp. (N. Tanaka & al. 080631)	*Potamogeton* sp. (N. Tanaka & al. 080662)
Characters								
Carpel number^a,b,c,d^		2	4	4	N/A	N/A	4	N/A
Leaf tip^a,b,c,d^		Round	Round	Acute	Round	Round	Acute	Round
Floating leaf vein^c,d^		11-21	11-21	9-13	13-18	10-12	14	9-13
Petiole length (Submerged leaves)^a^		Petioled	Unpetioled	N/A	Petioled	Petioled	Petioled	Petioled
Petiole length (Submerged leaves)^b^		1-200 mm	1-200 mm	2-70 mm	25-30 mm	40-50 mm	80-100 mm	25-30 mm
Petiole length (Submerged leaves)^d^		1.5-2.3 x length of blade	0.2-1.5 x length of blade	N/A	0.2-0.3 x length of blade	0.7-0.8 x length of blade	0.8-1.0 x length of blade	0.2-0.4 x length of blade
Petiole length (Floating leaves)^b^		80-260 mm	18-210 mm	N/A	45-85 mm	15-30 mm	85-120 mm	75-120 mm
Petiole length (Floating leaves)^c^		up to 400 mm	up to 200 mm	up to 200 mm				

**Table 2.** List of the GenBank accessions of *atpB*–*rbcL*, *rpl20*–*rps12*, *trnT*–*trnL*, *trnL*, *trnL*–*trnF*, and nrITS for ingroup and outgroup of *Potamogeton* species used in the phylogenetic analyses. Sequences obtained in the present study are shown in underline. Note that four Myanmar specimens are identified by DNA barcoding (see Discussion). Herbaria abbreviations: Forest Herbarium, Bangkok, Thailand = BKF; Wuhan Institute of Botany, Hubei, People's Republic of China = HIB; Kochi Prefectual Makino Botanical Garden, Kochi, Japan = MBK; Forest Research Institute, Pyinmana, Myanmar = RAF, The University of Tokyo Herbarium, Tokyo, Japan = TI; National Museum of Nature and Science, Tsukuba, Japan = TNS.

Accession	Locality	Voucher	*atpB*-*rbcL*	*rpl20*-*rps12*	*trnT*-*trnL*	*trnL*	*trnL*-*trnF*	nrITS
INGROUP								
*Potamogeton distinctus*								
	Japan	YI01686 (TNS)	AB871488	AB871498	AB744025	AB744013	AB744019	AB744007
	Thailand	YI01729 (BKF)	AB871490	AB871500	AB871505	AB871511	AB871517	AB871525
	Myanmar	N. Tanaka & al. 080061 (RAF, TI, MBK)	AB871483	AB871491	AB871501	AB871506	AB871512	AB871518
	Myanmar	N. Tanaka & al. 080657 (RAF, TI, MBK)	AB871485	AB871493	AB871503	AB871508	AB871514	AB871519
*Potamogeton nodosus*								
	Mexico	YI01195 (TNS)	AB871487	AB871497	AB871504	AB871510	AB871516	AB871524
*Potamogeton wrightii*								
	Japan	YI00048 (TNS)	AB206988	AB871495	AB695139	AB695131	AB695135	AB206991
Potamogeton ×malainoides								
	China	HDZY8 (HIB)	N/A	N/A	N/A	N/A	N/A	FJ956881
								FJ956882
	Myanmar	N. Tanaka & al. 080631 (RAF, TI, MBK)	AB871484	AB871492	AB871502	AB871507	AB871513	AB871520
								AB871521
*Potamogeton distinctus* × *Potamogeton nodosus*								
	China	HDZY5 (HIB)	N/A	N/A	N/A	N/A	N/A	FJ956875
								FJ956876
	China	HDZY6 (HIB)	N/A	N/A	N/A	N/A	N/A	FJ956877
								FJ956878
	China	HDZY7 (HIB)	N/A	N/A	N/A	N/A	N/A	FJ956879
								FJ956880
	Myanmar	N. Tanaka & al. 080662 (RAF, TI, MBK)	AB871486	AB871494	N/A	AB871509	AB871515	AB871522
								AB871523
OUTGROUP								
*Potamogeton perfoliatus*	Japan	YI01687 (TNS)	AB871489	AB871499	AB744026	AB744014	AB744020	AB744008
*Potamogeton lucens*	Japan	YI00049 (TNS)	AB206987	AB871496	AB695137	AB695129	AB695133	AB206990

**Table 3.** Comparison of the ITS sequences of the three broad-leaved long-petioled *Potamogeton* species and hybrids used in the phylogenetic analysis. Note that substitutions observed at 571 bp and 579 bp are due to apparent infra-specific variation in *Potamogeton nodosus*.

Taxon	nrITS									
	14	21	55	426	436	444	480	561	571	579
*Potamogeton distinctus*	T	C	T	C	A	G	T	G	G	T
*Potamogeton distinctus* × *Potamogeton nodosus*	T	C	T	C	A	G	T	G	G	T
	T	C	T	G	C	G	T	G	G	T
*Potamogeton nodosus* (Mexico)	T	C	T	G	C	G	T	G	A	C
*Potamogeton wrightii*	G	A	A	C	C	A	A	A	G	T
Potamogeton ×malainoides	T	C	T	C	C	A	T	G	G	T
	G	A	A	C	C	A	A	A	G	T

**Table 4.** Comparison of the *atpB*-*rbcL* and *trnT*-*trnL* sequences of the three broad-leaved long-petioled *Potamogeton* species and hybrids used in the phylogenetic analysis.

Taxon	*atpB*-*rbcL*			*trnT*-*trnL*	
	380-383	547	563	403-405	507-514
*Potamogeton distinctus*	ATTT	A	G	T (3)	A (8)
*Potamogeton nodosus*	ATTT	A	C	T (2)	A (8)
*Potamogeton wrightii*	------	C	G	T (2)	A (7)
